# From Cadaver to Clinic: Transforming First-Year Anatomy Education Through Early Ultrasound Integration

**DOI:** 10.7759/cureus.87013

**Published:** 2025-06-30

**Authors:** Garima Sehgal, Nikhil Aggarwal

**Affiliations:** 1 Anatomy, King George’s Medical University, Lucknow, IND; 2 Anatomy, Army College of Medical Sciences, New Delhi, IND

**Keywords:** anatomy education, cadaveric dissection, competency-based curriculum, medical imaging, ultrasound in education

## Abstract

Anatomy forms the foundational pillar of medical education, traditionally taught through cadaveric dissection. However, in light of evolving clinical demands and advancements in imaging technology, there is a growing need to enrich preclinical teaching methods. This article advocates for the structured integration of ultrasound into the first-year anatomy curriculum, in alignment with the National Medical Commission’s competency-based framework, which emphasizes early clinical exposure and hands-on learning. While cadaveric dissection cultivates spatial orientation and tactile skills, it lacks the dynamic representation of physiological processes essential in modern diagnostic practice. Ultrasound bridges this gap by offering real-time, radiation-free visualization of living anatomy, enabling students to correlate theoretical knowledge with functional observations such as cardiac motion, vascular flow, and visceral organ activity. For students, this integration fosters improved spatial understanding, early diagnostic reasoning, and enhanced retention through experiential learning. For example, visualizing the pulsating vessels on a color Doppler or the dynamic movement of abdominal organs during sonographic sessions makes anatomical learning more engaging and clinically meaningful. Such exposure equips students with foundational skills in image acquisition and interpretation, preparing them for future clinical encounters from their formative years. For educators, ultrasound promotes active learning strategies and interdepartmental collaboration, encouraging innovation in teaching methodologies. It also complements didactic lectures and dissection sessions, creating a hybrid model that strengthens knowledge transfer and long-term retention. From a healthcare perspective, early ultrasound training helps develop clinicians who are better prepared to identify normal and pathological findings, particularly in primary care and resource-limited settings. This aligns with the vision of producing Indian medical graduates capable of serving as first-contact physicians in the community. Furthermore, literature evidence, including systematic reviews, demonstrates improved academic performance, diagnostic confidence, and clinical preparedness among students trained with ultrasound-enhanced curricula. In conclusion, incorporating ultrasound into first-year anatomy education is not merely an enhancement but a necessary evolution. It addresses limitations of traditional methods, meets regulatory mandates, and directly benefits students, educators, and the healthcare system. This integrated approach ensures medical graduates are equipped with both foundational anatomical knowledge and the clinical competencies required in contemporary practice.

## Editorial

Anatomical knowledge forms the cornerstone of medical education, traditionally rooted in detailed cadaveric dissections. This time-honored method faces evolving challenges, including cadaver availability and the dynamic landscape of modern diagnostics. While this classical approach has profoundly shaped generations of clinicians, the current landscape demands educational methodologies that align more closely with evolving clinical practices and technological advances. As medical imaging, particularly ultrasound, computed tomography, and magnetic resonance imaging, becomes increasingly central to clinical practice, it is imperative that our foundational anatomy curriculum adapts to reflect this reality. The integration of imaging modalities, particularly ultrasound, into anatomy curricula represents not merely an educational enhancement but a necessary evolution to equip future physicians for modern clinical realities [1].

Historically, cadaveric dissection has enabled students to develop tactile and spatial understanding of human anatomy. Yet, reliance solely on this method faces significant challenges, including limited cadaver availability, ethical considerations, and logistical constraints. Furthermore, static cadaver specimens cannot convey dynamic physiological processes, such as blood flow, muscle contraction, or cardiac activity, all vital aspects for comprehending living anatomy [2]. Therefore, incorporating dynamic imaging techniques such as ultrasound offers a complementary, more clinically relevant educational experience.

Ultrasound imaging has emerged prominently among available modalities due to its unique advantages, such as portability and safety (being free of ionizing radiation). It provides real-time visualization of living anatomical structures, enabling students to directly observe functional processes in organs and tissues. Such an approach not only improves spatial understanding but also closely links anatomical learning to clinical diagnostic skills. Early ultrasound exposure facilitates foundational skill development in image acquisition, interpretation, and clinical correlation, preparing students comprehensively for their future roles as clinicians [2,3]. Recent research confirms the educational efficacy of integrating ultrasound early into the medical curriculum. For instance, a systematic review by Tarique et al. highlights substantial evidence that students trained in ultrasound exhibit superior knowledge retention, enhanced diagnostic confidence, and improved spatial understanding compared to peers taught exclusively through traditional methods [3]. Students gain the ability to correlate surface anatomy with internal structures and begin forming diagnostic reasoning skills that are crucial in clinical practice.

This proactive approach aligns perfectly with the National Medical Commission guidelines on competency-based education, which strongly emphasize early clinical exposure for first-year students [4]. By incorporating ultrasound from the outset, we are not just teaching anatomy; we are actively cultivating the diagnostic mindset and practical skills essential for future clinicians. These physicians of first contact will be trained from their formative years in the identification of the normal sonographic appearance of body tissues and organs. This foundational knowledge will be crucial for them to better diagnose abnormalities and pathologies, skills that can be further honed in their clinical years. This comprehensive training will equip them to recognize common medical and surgical conditions at primary and secondary-level health centers, enabling them to provide necessary guidance and timely referrals to the community. This serves a crucial purpose: to accomplish the goal of preparing Indian medical graduates as skilled clinicians to serve as a primary physician of first contact to the community. Students gain hands-on experience that bridges the gap between theoretical knowledge and real-world application, making their learning immediately applicable and significantly more engaging.

The practical benefits of ultrasound integration extend beyond improved anatomical understanding alone. Clinically, ultrasound has become an indispensable diagnostic tool in diverse medical specialties, ranging from emergency and critical care medicine to obstetrics and primary care settings [1]. Consequently, proficiency in ultrasound interpretation and application is increasingly viewed not as optional but as a fundamental clinical competency. Early and structured training prepares students effectively, instilling confidence and clinical relevance to their anatomical knowledge.

Moreover, ultrasound education resonates profoundly with contemporary educational philosophies promoting active and experiential learning. Incorporating ultrasound within the anatomy curriculum encourages student engagement through interactive sessions, enhancing their intrinsic motivation and reinforcing long-term retention. Christopher L. Moore and Joshua A. Copel emphasized that integrating point-of-care ultrasound in medical education significantly boosts students’ preparedness and clinical decision-making abilities, advocating strongly for its broader educational adoption [5].

Having personally observed the profound impacts of ultrasound integration through my role as in-charge of the Ultrasound Skills Lab in our department, I affirm its effectiveness. Every year, 15 students in Phase 3 MBBS are given the opportunity to take the two-week Ultrasound Elective course. Its goal is to impart fundamental knowledge and practical skills for using ultrasound to identify normal anatomical structures. This elective course aims to improve students’ procedural skills early in their medical careers, preparing them to incorporate ultrasound into their future clinical practice.

Interactive lectures, group discussions, demonstrations, and supervised hands-on activities are all a part of the teaching and learning sessions. Peer and self-directed learning are also highlighted as ways to promote both individual and group development. Students work with fundamental concepts such as image orientation, transducer handling, machine controls (knobology), and ultrasound principles. They also learn how to locate and distinguish different anatomical structures on ultrasound, as well as how to identify scan planes. Localization and identification of normal anatomical structures, such as muscles, nerves, and vessels, and abdominal organs, such as the liver, gallbladder, and kidneys, are the main areas discussed.

Regular daily involvement in supervised scanning sessions during the electives course resulted in improvement in probe handling, image acquisition, and anatomical structure identification. Students advanced from minimal familiarity to confidently performing targeted scans with growing independence over the course of the two weeks. Along with the practical experience, students also participated in structured reflections to evaluate their scanning experiences, identify areas for growth, and use the feedback to improve performance in the future.

Our experience with the sonographic anatomy electives session demonstrates that students rapidly develop clinical acumen when anatomy education incorporates ultrasound visualization [2,3]. They become adept not only at identifying anatomical structures but also at recognizing and interpreting clinically significant variations. Such competencies directly enhance their diagnostic reasoning abilities, thereby bridging the theoretical-practical divide effectively.

However, transitioning to ultrasound-enriched anatomy curricula is accompanied by challenges. Key obstacles include the initial financial investment, faculty training requirements, and curriculum restructuring. Financial considerations, although substantial, are balanced by long-term benefits, including reduced reliance on cadavers, improved student performance, and enhanced clinical readiness. Addressing faculty training is equally crucial, requiring dedicated professional development programs that empower anatomists to confidently teach basic ultrasound techniques. Strategic curriculum design ensures seamless integration, avoiding curricular overload by effectively blending traditional dissections with ultrasound-based sessions.

Successful implementation typically involves a hybrid educational approach. Traditional dissections provide essential tactile experience and anatomical appreciation, complemented by dynamic, real-time ultrasound sessions. Such an integrated model optimizes student learning outcomes, ensuring a balanced yet modern anatomical education framework. Encouraging collaborative teaching across anatomy, radiology, and clinical departments further enriches the educational experience, fostering multidisciplinary perspectives vital to clinical practice.

An interesting review by Tarique et al. provides strong empirical support for this integrated approach. It indicated that medical students taught with integrated ultrasound curricula not only perform better academically but also show higher clinical competence and greater preparedness for patient encounters [3]. These outcomes highlight the tangible benefits of early ultrasound education, underscoring its potential to transform anatomical education significantly.

The evolving landscape of healthcare necessitates dynamic adjustments in medical education. Integrating ultrasound into first-year anatomy curricula meets this necessity effectively. Early exposure to clinical imaging equips students with the foundational skills required for modern medical practice, cultivating diagnostic acumen from their earliest educational stages. Such a proactive approach ensures that graduates are well-prepared to meet contemporary clinical challenges with confidence and competence.

In conclusion, transitioning anatomy education from traditional cadaver-centric methods to a curriculum enriched by ultrasound integration is imperative. This approach does not diminish the historical value of cadaveric dissections; rather, it enhances it by incorporating dynamic, clinically relevant techniques essential for contemporary medical practice. Educational institutions must embrace this transformative shift to adequately prepare the next generation of clinicians, thereby ensuring comprehensive, relevant, and modern medical education aligned with clinical realities.
